# Rationally Designed Antimicrobial Peptides Are Potential Tools to Combat Devastating Bacteria and Fungi

**DOI:** 10.3390/ijms23116244

**Published:** 2022-06-02

**Authors:** Györgyi Váradi, László Galgóczy, Gábor K. Tóth

**Affiliations:** 1 Department of Medical Chemistry, Albert Szent-Györgyi Medical School, University of Szeged, Dóm tér 8, 6720 Szeged, Hungary; varadi.gyorgyi@med.u-szeged.hu; 2 Department of Biotechnology, Faculty of Science and Informatics, University of Szeged, Közép fasor 52, 6726 Szeged, Hungary; galgoczi@bio.u-szeged.hu; 3 Institute of Biochemistry, Biological Research Centre, Eötvös Loránd Research Network, Temesvári krt. 62, 6726 Szeged, Hungary; 4 MTA-SZTE Biomimetic Systems Research Group, Department of Medical Chemistry, University of Szeged, Dóm tér 8, 6720 Szeged, Hungary

The introduction of the first antibiotic (penicillin) by Sir Alexander Fleming in 1928 was a huge milestone in the treatment of infectious diseases. Unfortunately, after several years the development of antimicrobial resistance (AMR) became one of our most serious health threats. AMR is a global healthcare problem causing the death of nearly 700,000 people each year, and predictions indicate that this number may reach up to 10 million deaths annually by 2050 [1]. Recently the annual cost of AMR was estimated at EUR 1.5 billion in the EU and USD 5 billion in the USA. According to the Infectious Diseases Society of America (IDSA), the most worrying bacteria comprise the multidrug-resistant (MDR) ESKAPE pathogens *Enterococcus faecium*, *Staphylococcus aureus*, *Klebsiella pneumoniae*, *Acinetobacter baumannii*, and *Enterobacter* species, which together cause the majority of hospital infections [2]. According to a recent Lancet publication, “There were an estimated 4.95 million deaths associated with bacterial AMR in 2019, including 1.27 million deaths attributable to bacterial AMR” [3]. Nowadays, fungal infections in humans are considered as a silent global crisis. Human pathogenic fungi cause approximately 1.7 million deaths per year, and they represent an important threat to public health as a consequence of fast drug resistance spreading, an emerging number of MDR strains, and the lack of effective antifungal agents [4]. These scaremongering resistances reflected in the numbers mentioned above indicate the continuously increasing demand for new types of antibacterial [5] and antifungal [6] substances. AMR is a serious problem not only in medicine but also in agriculture. Crop losses directly due to pesticide resistance are worth USD ~1.5 billion per year just in the USA [7]. Because of enormous crop losses worldwide due to pesticide-resistant plant pathogenic bacteria and fungi, there is a growing interest in the development of novel antibacterial and antifungal strategies in agriculture [8].

Natural proteins and peptides with antimicrobial activities (AMPs) are promising candidates to overcome the above-mentioned drug-resistance crises [9]. Several features make them promising drug candidates; among them, the most important ones are the standard synthetic protocols, rapid killing kinetics, a broad range of antimicrobial action, and low potential for resistance development. However, several factors limit their direct medical and agricultural applications, e.g., the high cost of production, short half-life, limited storage, occasional narrow antimicrobial spectrum, poor pharmacokinetic and pharmacodynamic profiles, and potentially detrimental effects on the host [10,11]. Based on structural characteristics, the more than 3000 AMPs currently known can be divided into two groups: linear, usually helical peptides (e.g., cathelicidins) and cysteine-rich, multiple disulfide bridges containing peptides (e.g., defensins, nodule-specific cysteine-rich plant (NCR) peptides, and fungi originated peptides) [12]. During the search for new, effective AMPs, several problems need to be solved. Some of these are the elimination of toxic side effects (e.g., hemolysis), the improvement of minimum inhibitory concentration and minimum bactericidal/fungicidal concentration values, and increasing the chemical and biological stability of the peptides. There are many different tools in the hands of researchers to unravel these questions. The examination of the molecular structures of AMPs, investigation of structure-activity relationships, design, chemical synthesis, and testing of smaller fragments or modified analogs of naturally occurring peptides, and the application of chimeras and artificial building blocks are among those strategies that are used to gain valuable pieces of information about AMPs and to improve access to more feasible antimicrobial agents. Our previous studies with defensin-like NCR peptides [13,14] and γ-core peptide derivatives of antifungal proteins [15,16,17,18] already provide examples of the rational modifications of AMPs in these aspects.

Legume plants from the inverted repeat-lacking clade represent a remarkable source of antimicrobials secreting over 700 NCR peptides [19,20]. NCR peptides contain four or six cysteines, and the cysteines are crucial for their biological activity [21]. The smallest member of the family, the 24-mer NCR247 [22], provides a good example of improving the antimicrobial activity of naturally occurring peptides by modifications of the original sequence. We designed and synthesized shorter fragments and chimeric derivatives of NCR247 [13]. The C terminal half of the peptide partially retained antibacterial activity, while the fusion of this 12-mer with a truncated mastoparan sequence increased the bactericidal effect and altered the antibacterial spectrum. The most potent derivative was found to be much more effective than most classical antibiotics with µM concentration. Some NCR peptides demonstrate not only antibacterial but also antifungal effects. We showed that both the N- and C-terminal regions of NCR335, as well as the C-terminal sequence of NCR169, possessed anti-*Candida* activity [14].

A valuable source of natural antifungal compounds is the group of defensin-like antifungal proteins from filamentous fungi. An evolutionarily conserved region of these antifungal proteins is the so-called γ-core motif consisting of the amino acid sequence GXCX_3–9_C. The 14 amino acid long peptide designed on the γ-core of the *Penicillium chrysogenum* antifungal protein (PAF) showed growth inhibition activity against the opportunistic human pathogen yeast, *Candida albicans* [15]. Increasing the net charge and hydrophilicity by amino acid substitutions enhanced the antifungal effect of the peptide. Moreover, none of the peptides caused hemolysis on erythrocytes. Both the optimized (more positively charged and more hydrophilic) γ-core peptide and the full-length protein containing this modified γ-core were found to be antifungal active against important plant pathogenic ascomycetes. The γ-core modification of the native PAF altered the antifungal spectrum of the protein against this group of fungi [16]. Similarly, the γ-core peptide of *Neosartorya fischeri* antifungal protein (NFAP), as well as its analogs, inhibited the growth of agriculturally relevant filamentous ascomycetes in vitro while they were not cytotoxic or hemolytic [17]. The synergistic activity of NFAP and its optimized γ-core peptide in tomato plant biocontrol experiments against the necrotrophic plant pathogen *Botrytis cinerea* infection was proved [18]. From these investigations, it could be concluded that a short peptide spanning the γ-core of PAF or NFAP and their appropriately substituted analogs could possess antifungal activity against both clinically and agriculturally relevant fungi.

The present Special Issue provides some attractive solutions for modifications of naturally occurring AMPs to improve their potential for medical or agricultural applications. C-terminal lipidation of BAC7(1-16), a short proline-rich AMP altered its function and mode of action [23]. The lipidated analogs did not select resistant mutants in *Escherichia coli* after repeated exposure to sub-MIC concentrations. In the case of three 15-mer human mucin 7 (MUC7) peptides, a correlation between lipophilicity, the presence of metal ions, and antimicrobial activity against Gram-positive and negative bacteria, as well as fungi, was proven [24]. Since lipidation has an important role in improving the pharmacokinetic and pharmacodynamic characteristics of natural AMPs, a model lipopeptide was designed and synthesized to examine the effect of the hydrocarbon chain’s length on biological functions and mode of action [25]. Another study demonstrated that octominin, a synthetic anticandidal peptide from a defense protein from *Octopus minor* [26] exhibited significant antibacterial and antibiofilm activities against the multidrug-resistant *A. baumannii*, while it was not hemolytic and did not change the viability of mammalian cells [27].

In summary, the “Peptide Antimicrobial Agents” Special Issues and the other papers we have cited here introduce promising candidates for the design of new antimicrobial agents. We hope that the knowledge collected here could contribute to the alleviation of damages caused by bacteria and fungi.
